# Induction of multiple pleiotropic drug resistance genes in yeast engineered to produce an increased level of anti-malarial drug precursor, artemisinic acid

**DOI:** 10.1186/1472-6750-8-83

**Published:** 2008-11-04

**Authors:** Dae-Kyun Ro, Mario Ouellet, Eric M Paradise, Helcio Burd, Diana Eng, Chris J Paddon, Jack D Newman, Jay D Keasling

**Affiliations:** 1 Department of Biological Sciences, University of Calgary, Calgary, T2N 1N4, Canada; 2 California Institute of Quantitative Biomedical Research, University of California, Berkeley, USA; 3 Department of Chemical Engineering, University of California, Berkeley, 94720, USA; 4 Amyris Biotechnologies, Emeryville, USA; 5 Department of Bioengineering, University of California, Berkeley, USA; 6 Physical Biosciences Division, Lawrence Berkeley National Laboratory, Berkeley, USA

## Abstract

**Background:**

Due to the global occurrence of multi-drug-resistant malarial parasites (*Plasmodium falciparum*), the anti-malarial drug most effective against malaria is artemisinin, a natural product (sesquiterpene lactone endoperoxide) extracted from sweet wormwood (*Artemisia annua*). However, artemisinin is in short supply and unaffordable to most malaria patients. Artemisinin can be semi-synthesized from its precursor artemisinic acid, which can be synthesized from simple sugars using microorganisms genetically engineered with genes from *A. annua*. In order to develop an industrially competent yeast strain, detailed analyses of microbial physiology and development of gene expression strategies are required.

**Results:**

Three plant genes coding for amorphadiene synthase, amorphadiene oxidase (*AMO *or *CYP71AV1*), and cytochrome P450 reductase, which in concert divert carbon flux from farnesyl diphosphate to artemisinic acid, were expressed from a single plasmid. The artemisinic acid production in the engineered yeast reached 250 μg mL^-1 ^in shake-flask cultures and 1 g L^-1 ^in bio-reactors with the use of *Leu2d *selection marker and appropriate medium formulation. When plasmid stability was measured, the yeast strain synthesizing amorphadiene alone maintained the plasmid in 84% of the cells, whereas the yeast strain synthesizing artemisinic acid showed poor plasmid stability. Inactivation of AMO by a point-mutation restored the high plasmid stability, indicating that the low plasmid stability is not caused by production of the AMO protein but by artemisinic acid synthesis or accumulation. Semi-quantitative reverse-transcriptase (RT)-PCR and quantitative real time-PCR consistently showed that pleiotropic drug resistance (*PDR*) genes, belonging to the family of ATP-Binding Cassette (ABC) transporter, were massively induced in the yeast strain producing artemisinic acid, relative to the yeast strain producing the hydrocarbon amorphadiene alone. Global transcriptional analysis by yeast microarray further demonstrated that the induction of drug-resistant genes such as ABC transporters and major facilitator superfamily (MSF) genes is the primary cellular stress-response; in addition, oxidative and osmotic stress responses were observed in the engineered yeast.

**Conclusion:**

The data presented here suggest that the engineered yeast producing artemisinic acid suffers oxidative and drug-associated stresses. The use of plant-derived transporters and optimizing AMO activity may improve the yield of artemisinic acid production in the engineered yeast.

## Background

Terpenoids (or isoprenoids) are a large and diverse class of natural products derived from five-carbon building unit, isopentenyl diphosphate (IPP) [1,2]. The central precursor IPP and its isomer (dimethyl allyl diphosphate, DMAPP) are converted to the 10-carbon geranyl diphosphate (GPP), the 15-carbon farnesyl diphosphate (FPP), and the 20-carbon geranylgeranyl diphosphate (GGPP) by the condensation reactions of GPP, FPP, and GGPP synthase, respectively. At the entry-point of terpenoid biosynthesis, the IPP and its derivatives (i.e., GPP, FPP, and GGPP) are transformed to hundreds of unique hydrocarbon olefins by terpene synthases via carbocation intermediates [3]. These terpene backbones are then decorated by modifying enzymes such as cytochrome P450 monooxygenase (P450), oxidoreductase, and other transferase enzymes that provide various functional moieties (e.g., methyl, acetyl, phenolic groups).

In primary metabolism, terpenoids are indispensable components for various physiological processes, such as respiration (ubiquinone), photosynthesis (plastoquinone), membrane fluidity (cholesterol), and intracellular signaling cascades (protein prenylation). Terpenoid metabolism is also responsible for creating a wide array of related, yet chemically distinct natural products, which play important roles in interactions among organisms and defense mechanisms against biotic stresses [4,5]. Many of these terpenoid natural products have found use as pharmaceuticals (e.g., taxol as an anti-cancer drug), nutraceuticals (e.g., lycopene as an anti-oxidant), aromas and flavors (e.g., nootkatone as an aroma), and industrial chemicals (e.g., natural rubber). The transformation of IPP and its related derivatives to highly complex terpenoids has been an area of active biochemical and bio-engineering studies [6].

The pharmaceutical, chemical, and food industries that supply terpenoid commodities face two critical issues. First, the chemical complexities of terpenoids hinder economic chemical synthesis of terpenoids. To date, the supply of many terpenoid compounds still depends on the isolation of natural terpenoids or the pathway intermediates from plant sources. Second, chemical intermediates and solvents required for the organic chemical synthesis of terpenoids are often petroleum-derived chemicals whose availability is finite. To circumvent these problems, current biotechnological efforts have been focused on devising novel biological processes to manufacture complex terpenoids using enzymes and engineered microbial platforms. One example of biological manufacturing of terpenoids is the production of the anti-malarial drug artemisinin precursor, artemisinic acid, using recombinant enzymes in microbial platforms [7,8].

Artemisinin is a sesquiterpene lactone endoperoxide extracted from the medicinal plant, sweet wormwood (*Artemisia annua*). Artemisinin is a potent anti-malarial drug whose mode of action in curing malaria is proposed to include inhibition of the SERCA (Sarco/Endoplasmic Reticulum Ca^2+^-ATPase) activity of *Plasmodium falciparum *[9]. Artemisinin Combination Therapy (ACT) has been recommended as the first-line medication to patients living in areas where multi-drug resistant strains of *Plasmodium spp*. are endemic [10,11]. However, the lack of sufficient raw material, artemisinin, and the cost associated with the drug's manufacture have limited the supply of ACT to malaria sufferers in the Developing World [12]. At this moment, scientific effort is being directed to develop a biological method to supply sufficient and reliable quantities of artemisinic acid, a direct precursor of artemisinin. The introduction and optimization of the mevalonate pathway in *E. coli *increased production of FPP to 0.5 gram L^-1 ^as measured by amorphadiene production [13]. Similar flux-enhancement in *S. cerevisiae *increased amorphadiene production to 150 mg L^-1 ^[7,14]. Expression of amorphadiene synthase (*ADS*), amorphadiene C12 oxidase (*AMO *or *CYP71AV1*), and its redox partner cytochrome P450 reductase (*CPR*) in the flux-enhanced *E. coli *or *S. cerevisiae *demonstrated that more than 100 mg artemisinic acid L^-1 ^culture can be produced in both platforms at a laboratory scale [7,8]. The efficient conversion of amorphadiene to artemisinic acid by *S. cerevisiae *expressing the ER-bound AMO demonstrated it to be an ideal microbial platform to functionally express genes encoding membrane-bound enzymes.

Following up from the report of artemisinic acid manufacture in the engineered yeast [7], we evaluated and further improved the engineered yeast as a microbial host to produce the drug precursor artemisinic acid. We show here that the production of artemisinic acid can be increased by employing a high-copy plasmid system. We further assessed the response of the engineered yeast to artemisinic acid synthesis and accumulation by analyzing the global transcript profile and by conducting targeted gene expression analysis of multi-drug resistant genes. The data demonstrates the potential use of the engineered yeast to produce drug precursors and also suggests molecular strategies to be employed to further improve the artemisinic acid production levels.

## Results

### Low plasmid stability in yeast strain engineered to produce artemisinic acid

**Figure 1:**
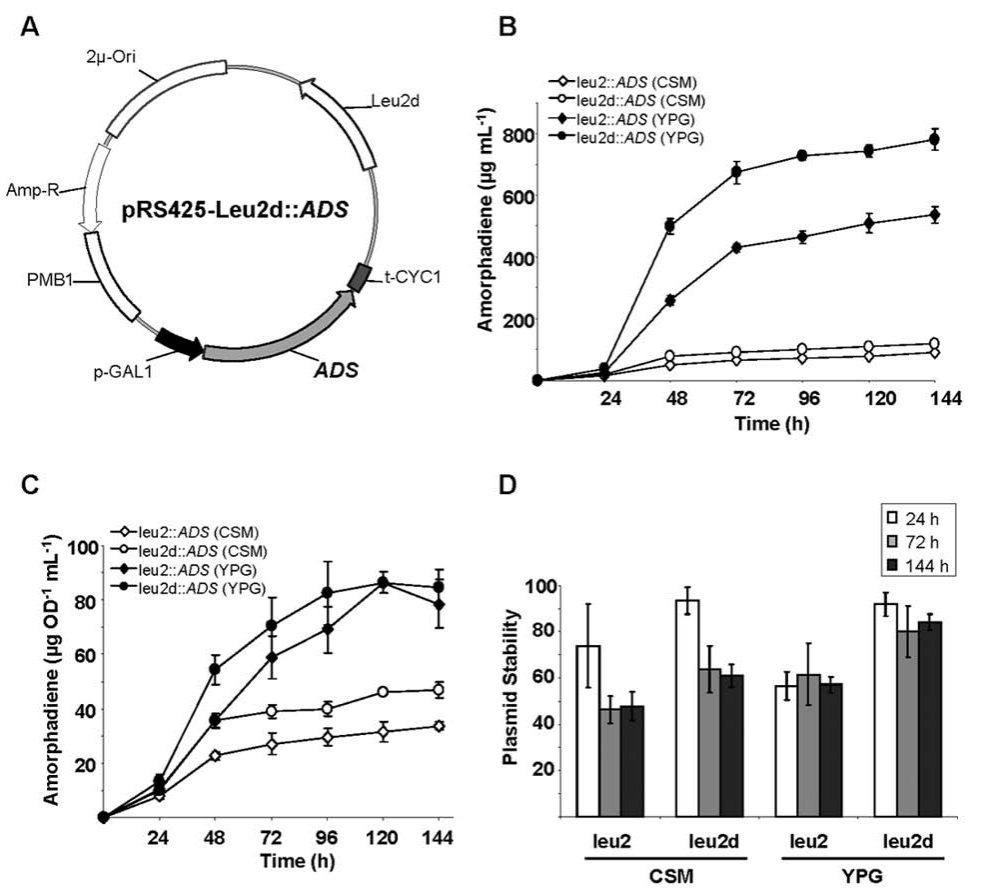
**Amorphadiene production in the engineered yeast strain**. (A) Plasmid harboring *ADS *under control of the *Gal1 *promoter used to produce amorphadiene in EPY300. Total (B) and specific (C) amorphadiene production from EPY300 expressing *ADS *on either pRS425-leu2 or pRS425-leu2d cultured in either CSM or YPG medium. Circles and diamonds indicate the leu2d and leu2 selection markers, respectively. Unfilled symbols indicate selective, minimal medium (CSM), and filled symbols indicate non-selective, rich medium (YPG). (D) Plasmid stabilities of yeast harboring pRS425-leu2::*ADS *or pRS425-leu2d::*ADS *grown in either CSM or YPG medium at 24, 72, and 144 hr post-induction. Data are mean ± standard deviation from three replicate experiments.

**Figure 2:**
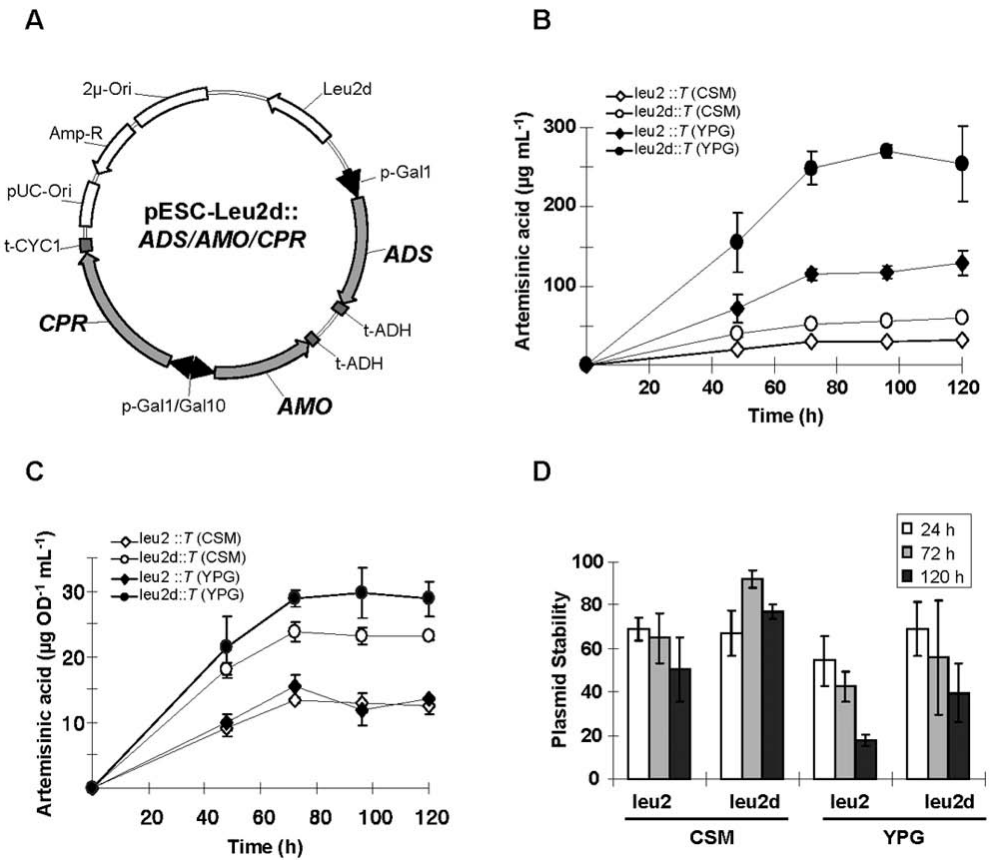
**Artemisinic acid production by the engineered yeast strain**. (A) Plasmid encoding *ADS*, *AMO*, and *CPR *under control of either the *Gal1 *or *Gal10 *promoter used to produce artemisinic acid by EPY300. Total (B) and specific (C) artemisinic acid production from EPY300 strain expressing *ADS, AMO*, and *CPR *from either pESC-leu2 or pESC-leu2d cultured in either CSM or YPG medium. *T *indicates triple genes (*ADS*, *AMO*, and *CPR*). Circles and diamonds indicate leu2d and leu2 selection markers. Unfilled symbols indicate selective, minimal medium (CSM), and filled symbols indicate non-selective, rich medium (YPG). (D) Plasmid stabilities of yeast harboring either pRS425-leu2::*ADS/AMO/CPR *or pRS425-leu2d::*ADS/AMO/CPR *grown in either CSM or YPG medium at 24, 72, and 120-hr post-induction.

Artemisinic acid is a key precursor for the anti-malarial drug, artemisinin. We have previously reported an engineered yeast strain harboring two plasmids that can synthesize ~100 mg artemisinic acid L^-1 ^culture in a bioreactor [7]. In the previous study, expression of the three plant genes was achieved using the *pRS425-Leu *plasmid for amorphadiene synthase (*ADS*) expression and using the *pESC-Ura *plasmid for amorphadiene oxidase (*AMO*) and cytochrome P450 reductase (*CPR*) expressions. The plasmid stability of the engineered yeast was evaluated after culturing the yeast strain in galactose selective medium. After culturing for 120 hr, only 28 ± 10% (mean ± SD, n = 3) of the engineered yeast contained both *pRS425-Leu::ADS *and *pESC-Ura::AMO/CPR *plasmids, indicating that 72% of the yeast cells lost the leucine, uracil, or both selectable markers. This result led us to systematically examine the plasmid stability of engineered yeast strains with the aim of devising methods to increase the yield of artemisinic acid production. We analyzed the production of amorphadiene and artemisinic acid separately by constructing a single vector encoding either *ADS *or *ADS/AMO/CPR *(Figure 1A and Figure 2A).

### Improved production of amorphadiene in engineered yeast

The *Leu2d *allele contains a deleted promoter, leaving only 29-bp of the *Leu2 *promoter region in the *Leu2 *expression cassette. To compensate for the weakened *Leu2 *expression, *S. cerevisiae *selects for significantly higher plasmid copy number [15]. The *ADS *gene was expressed in *pRS425-Leu2 *(EYP224) or in the newly constructed *pRS425-Leu2d *plasmid backbone (EPY230, Figure 1A) using selective (CSM) or non-selective medium (YPG). As amorphadiene is volatile, it was collected by overlaying dodecane in the yeast culture.

Amorphadiene dissolved in the dodecane layer was quantified by gas-chromatography. In the selective medium, changing expression of *ADS *from *pRS425-Leu2 *to *pRS425-Leu2d *increased amorphadiene production 1.3 fold (from 90 ± 3 μg mL^-1 ^to 120 ± 4 μg mL^-1^), whereas changing the culture medium from selective to non-selective rich medium using the *Leu2 *plasmid (*pRS425-Leu2*) increased amorphadiene production 5.9 fold (Figure 1B). Expression of *ADS *from *pRS425-Leu2d *in non-selective medium increased amorphadiene production by 8.6-fold (781 ± 34 μg mL^-1 ^culture). Specific amorphadiene production (i.e., amorphadiene production per OD unit in a given time) closely followed the time-course production pattern for total amorphadiene, although the production difference in non-selective medium was marginal (Figure 1C).

The stability of *pRS425-Leu2::ADS *plasmid in either selective or non-selective medium was not significantly different, exhibiting more than 50% plasmid stability at 144-hr post-induction in all conditions (Figure 1D). Interestingly, the stability of *pRS425-Leu2d::ADS *in non-selective medium was very high (84% ± 4) even at 144-hr post-induction. The high stability of *pRS425-Leu2d::ADS *implies that the *Leu2d *selectable marker enables yeast to maintain high plasmid stability in non-selective medium.

### Improved production of artemisinic acid in engineered yeast

Plasmids containing three plant genes (*ADS, AMO, CPR*) for artemisinic acid biosynthesis were constructed in *pESC-Leu2 *or *pESC-Leu2d *(Figure 2A). These two plasmids are referred to as *pESC-Leu2-T *(triple genes) or *pESC-Leu2d-T*. Prior to testing the artemisinic acid production by these two plasmids, the expression of *ADS *in the *pRS425-Leu2d *and *pESC-Leu2d *backbones was compared to ensure that *ADS *is expressed similarly in the two different plasmid backbones. The engineered yeast strain harboring either *pRS425-Leu2d*::*ADS *(EPY230; see Table 1) or *pESC-Leu2d::ADS *(EPY306) routinely produced 800 μg amorphadiene mL^-1 ^culture. There was no difference in their plasmid stabilities (85% ± 3 for *pRS425-Leu2d::ADS *and 90% ± 10 for *pESC-Leu2d::ADS*; n = 3). Thus, *ADS *expression was not affected by the type of plasmid backbones used.

**Table 1:** Strain names and genotypes

**Strains**	**Genotype**	**Plasmid**	**References**
BY4742	*MATαα his3Δ1 leu2Δ0 lys2Δ0 ura3Δ0*	none	41
EPY224	*MATαα his3Δ1 leu2Δ0 lys2Δ0 ura3Δ0 *P*GAL1-tHMGR *P*GAL1-upc2-1 erg9*::*PMET3-ERG9 *P*GAL1-tHMGR *P*GAL1-ERG20*	pRS425-Leu::*ADS*	7
EPY300	*MATαα his3Δ1 leu2Δ0 lys2Δ0 ura3Δ0 *P*GAL1-tHMGR *P*GAL1-upc2-1 erg9*::*PMET3-ERG9 *P*GAL1-tHMGR *P*GAL1-ERG20*	none	7
EPY230	EPY300	pRS425-Leu2d::*ADS*	This study
EPY305	EPY300	pESC-Leu:: *ADS/AMO/CPR*	This study
EPY306	EPY300	pESC-Leu2d::*ADS*	This study
EPY307	EPY300	pESC-Leu2d:: *ADS/AMO/CPR*	This study
EPY309	EPY300	pESC-Leu2d:: *ADS/AMO-KO/CPR*	This study
EPY330	*lys2Δ0 ura3Δ0 *repaired EPY300	pESC-Leu2d:: *ADS/AMO/CPR*	This study
EPY338	*lys2Δ0 ura3Δ0 *repaired EPY300	pESC-Leu2d:: *ADS/AMO-KO/CPR*	This study

To evaluate artemisinic acid production, the EPY300 strain was transformed either with *pESC-Leu2-T *(creating strain EPY305) or *pESC-Leu2d-T *(creating strain EPY307). EPY305 produced 31 ± 3 mg L^-1 ^(n = 3) artemisinic acid, which is comparable to the level of artemisinic acid produced by the strain harboring the two-plasmid system [7]. Changing the selection marker from *Leu2 *to *Leu2d *increased artemisinic acid production by 1.9-fold in selective medium, whereas changing the medium from selective to non-selective using the *Leu2 *plasmid increased artemisinic acid production by 4.1-fold (Figure 2B). The combination of the *Leu2d *selection marker and non-selective, rich medium increased the production of artemisinic acid by 8.1 fold. As a result, the final titer reached 254 ± 48 mg L^-1^culture (Figure 2B). The production yield of amorphadiene and artemisinic acid (Figure 1B and 2B) indicates that 32% of the amorphadiene was oxidized by *AMO *and *CPR *in the engineered yeast.

The time course data of specific artemisinic acid production of the triple gene-expressing yeasts were clearly different from those of amorphadiene production of the *ADS*-expressing yeasts (Figure 2C). The yeast strain expressing the three genes from *pESC-Leu2 *(EPY305) in non-selective medium showed greatly decreased specific productivity (~10 μg OD^-1 ^mL^-1^). Thus, without selection, the three plant genes need to be expressed from the high-copy *pESC-Leu2d *plasmid in order to maintain high specific productivity.

Plasmid stabilities of the triple-gene expression vectors were significantly different from those of *ADS*-expressing plasmids (Figure 2D). Plasmid stability of *pESC-Leu2-T *in selective medium (50 ± 15%) was 2.8-fold higher than that in non-selective medium (18 ± 3%) at 120-hr post-induction. Similarly, the plasmid stability of *pESC-Leu2d-T *in selective medium (77 ± 3%) was 1.9-fold higher than that in non-selective medium (40 ± 14%). These results showed that engineered yeasts lose the plasmids coding for the three plant genes when grown in non-selective rich medium and that the *Leu2d *selection marker is required for yeast to retain plasmids. The low plasmid stability for yeast expressing the three plant genes from *pESC-Leu2 *in non-selective medium is consistent with the significantly decreased specific productivity of the same strain (Figure 2C).

In order to evaluate the potential use of the yeast strain for industrial fermentation purposes, the two auxotropic markers (*lys2Δ0 ura3Δ0*) were repaired, and the resulting yeast strain (EPY330) was cultured in a fed-batch culture in a bio-reactor. Without culture optimization, the yeast strain produced 1.06 g artemisinic acid L^-1 ^after culturing for 144 hr (see Materials and Methods for details). These data strongly suggest the industrial potential of this yeast strain for artemisinic acid production.

### Restoration of high plasmid stability by point-mutation of *AMO*

**Figure 3:**
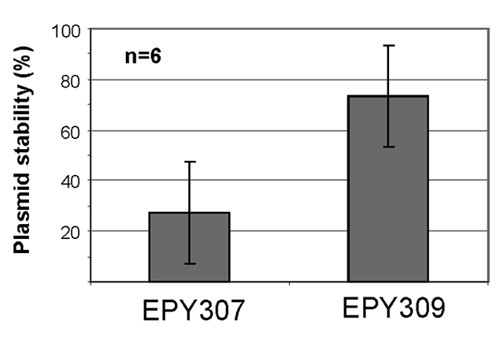
**Plasmid stabilities of transgenic yeast strains**. EPY307 harbors *pESC-Leu2d-T *plasmid and EPY309 harbors *pESC-Leu2d-TKO*, which carries *AMO *inactivated by the C439G point mutation.

Apparently, the plasmid stabilities were not affected by *ADS *gene expression and amorphadiene production (Figure 1D). However, a comparison of yeast strains producing amorphadiene to those producing artemisinic acid showed that plasmid stabilities were affected by *AMO *and *CPR *expression. To distinguish whether the plasmid loss was due to the high expression of membrane-bound AMO or due to the high level of artemisinic acid production, a point mutation (Cys to Gly at the 439th residue) was introduced into AMO at the cysteine residue that is conserved in all P450 enzymes. This *AMO *knock-out of *pESC-Leu2d-T *is referred to as *pESC-Leu2d-TKO*. GC-MS analysis confirmed that the engineered yeast strain containing the *pESC-Leu2d-TKO *(EYP309) did not produce artemisinic acid but produced a high level of amorphadiene, indicating that the C439G point mutation completely abolished the catalytic activity of *AMO *(data not shown). Production of the AMO protein in EPY309 was also verified by Western-blot using an anti-AMO polyclonal antibody (data not shown). Together, these data demonstrate that EPY309 is producing an inactive AMO protein. EPY300 was transformed with either *pESC-Leu2d-T *(EPY307) or *pESC-Leud2-TKO *(EPY309), and the plasmid stabilities of these two strains were measured after culturing at 96-hr in non-selective medium. The stability of *pESC-Leu2d-T *(28 ± 20%) was comparable to the previous data, whereas the stability of *pESC-Leu2d-TKO *was 74 ± 20% (Figure 3). This plasmid stability is much closer to that of the plasmid expressing *ADS *alone (Figure 1D). Based on these data, we concluded that artemisinic acid synthesis was the cause for the low plasmid stability.

### Induction of ABC transporter by endogenous production of artemisinic acid

**Figure 4:**
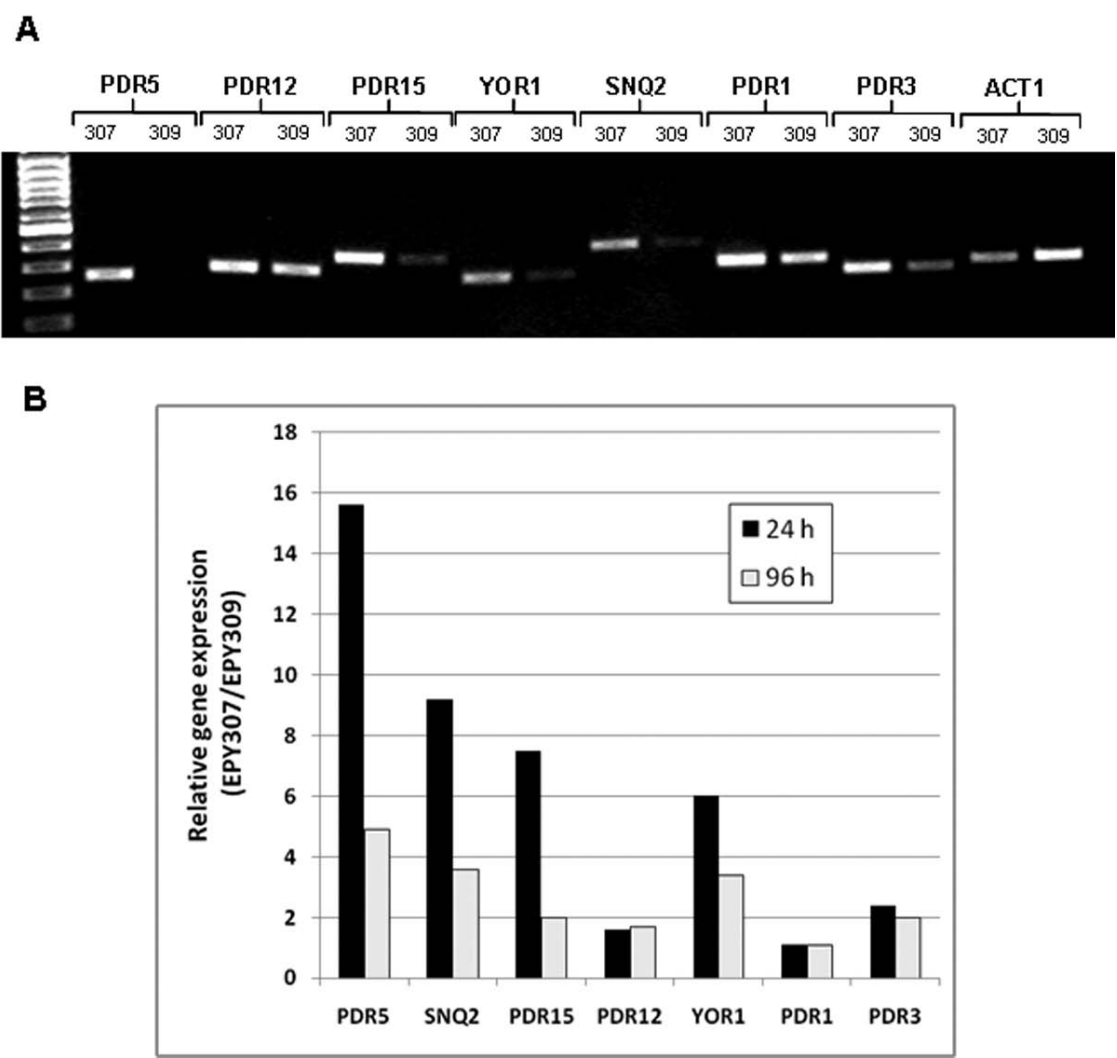
**Reverse-transcriptase PCR analysis of selected genes involved in multiple pleiotropic drug resistance**. (A) Semi-quantitative RT-PCR conducted with RNA extracted after 24 hr of induction from EPY307 (artemisinic acid producer) and EPY309 (amorphadiene producer). Number of PCR Cycles used: ACT1: 23; PDR5, PDR15, SNQ2: 24; YOR1:27; PDR1:28; PDR3, PDR12:30. (B) Quantitative real-time RT-PCR conducted with samples harvested at 24 and 96 h post-induction.

The two engineered yeast strains (EPY307 and EPY309) have a single nucleotide difference in their plasmids, which results in the different oxidation level of the terpenoid product (i.e., amorphadiene versus artemisinic acid). Considering the neutrality of the cytosol and a predicted pKa of 4.34 ± 0.11 for artemisinic acid [Scifinder data; calculated using Advanced Chemistry Development  Software V8.14 for Solaris], it is likely that artemisinic acid is present as an ionized form in the yeast cytosol, and hence active yeast transporters might be involved in transporting the polar artemisinic acid out of the cell to lessen its potential toxicity. To examine if the cellular stresses caused by a high level of oxidized terpenoids can evoke the induction of genes involved in pleiotropic drug resistance (PDR), the transcription of five yeast ATP-Binding Cassette (ABC) transporters (*PDR5, PDR12, PDR15, YOR1*, and *SNQ2*) and two known transcription factors (*PDR1 *and *PDR3*) for the PDR response were compared by semi-quantitative reverse transcriptase (RT)-PCR. A remarkable transcriptional induction of *PDR5 *was found in the yeast strain producing artemisinic acid relative to the strain expressing the inactivated AMO (Figure 4A). Expression of three other ABC transporters (*PDR15*, *YOR1*, and *SNQ2) *was also significantly induced in the yeast strain producing artemisinic acid relative to the yeast strain containing the inactivated *AMO*; induction of *PDR12 *expression was insignificant. In addition, up-regulated expression of the *PDR3 *transcription factor was noticeable after 24 hrs, while that of *PDR1 *was indiscernible.

In order to quantify the magnitude of induction, the transcripts of these genes were analyzed by quantitative real-time RT-PCR. At 24 hrs post-induction, *PDR5 *was induced by 15.6 fold; *PDR15*, *YOR1 *and *SNQ2 *were induced 6 to 9 fold; and *PDR3 *was induced by 2.4 fold (Figure 4B). Although expression induction of these genes was attenuated at 96 hrs, significant expression was still detectable. These data were consistent with and corroborate those obtained by semi-quantitative RT-PCR. Relative transcriptional analyses of yeast ABC transporters clearly demonstrated that one of the primary cellular responses caused by *de novo *artemisinic acid synthesis in engineered yeast is rapid transcriptional activation of various ABC transporters.

### Global transcriptional analysis by microarray

Up-regulation of PDR transporter expression suggests that *in vivo *production of artemisinic acid triggers a stress adaptation response in yeast. In order to further characterize the stress responses accompanying *AMO *activity and artemisinic acid production, we compared transcript profiles of an artemisinic acid-producing strain (EPY330) and that of a strain expressing the inactivated *AMO *(EPY338). Expression of the triple-gene constructs in five independent transformants for each strain (active and inactive AMO) were induced and harvested at 24, 48 and 72 hours after induction.

**Figure 5:**
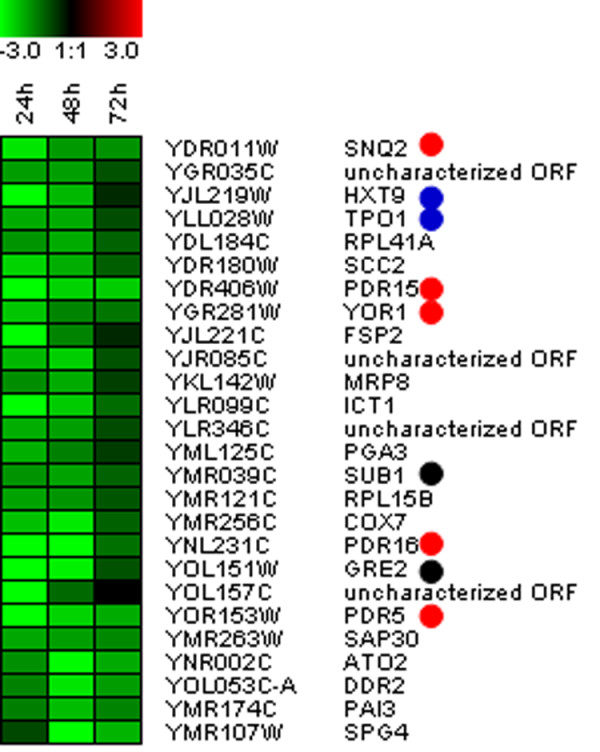
**Heat-map representation of some up-regulated yeast genes identified by microarray analysis**. Cluster 6 of k-mean clustering (k = 11) result showing some of the most strongly up-regulated genes observed in this study. Green color indicates the relative induction level (log2 ratio scale). *S. cerevisiae *open reading frame (ORF) and gene names are indicated. Red, blue, and black dots indicate genes for ATP-binding-cassette (ABC) superfamily, major facilitator superfamily (MFS), and oxidative stresses, respectively.

A total of 2,156 genes were found to be significantly differentially expressed in at least one experimental time point, of which 488 were up-regulated and 256 were down-regulated in the producer strain by more than 2-fold in at least one time point (see additional file 1 for a complete list of genes; the microarray data set was deposited at the Gene Expression Omnibus database, GEO Accession Number: GSE11620). Not unexpectedly, several known or putative PDR transporters (*PDR5, PDR10, PDR11, PDR12, PDR15, PDR16, PDR17, YOR1 and SNQ2*) were found among the differentially expressed genes, some of which are among the genes with the strongest up-regulation observed (Figure 5). These results are generally very similar to the results obtained by quantitative PCR for these genes, although *PDR12 *was found to be significantly up-regulated (1.9-fold) by microarrays at 72 h post-induction. Several transporters of the major facilitator superfamily (MFS) were also strongly up-regulated in the artemisinic acid-producing strain, including transporters associated with multiple drug resistance (*SGE1, DTR1, YCL073C, TPO1, TPO4*) and the hexose transporters (*HXT1, HXT9 *and *HXT11*), which have been shown to be transcriptionally controlled by PDR transcription factors Pdr1p and Pdr3p [16,17]. This important and widespread up-regulation of transcription of drug transporters often persisted at least 72 hr after induction. A number of genes in the gene ontology categories "response to drugs" or "response to toxin" (*CCR4, RIB1, PPA1, PRM7, MAC1, YLR056C, AAD6, AAD10, RTA1, YLR346C, YML131W, GTT2, GRE2*) were also strongly up-regulated.

The T-profiler software [18] and the YEASTRACT database [19] were used to interpret the gene expression profiles at the 24-hr time point by finding consensus motifs upstream of significantly represented groups of genes and associated transcription factors. Three different upstream regulatory consensus motifs were predicted to have a significant impact in the response to artemisinic acid production: TCCGYGGR, which can be bound by the drugs and stress -responsive transcription factors Pdr3p, Pdr8p, Yrr1p and Hsf1p; CCCCT, which can be bound by the drugs and stress-responsive transcription factor Msn2p/Msn4p; and YYACCCG, which can be bound by Reb1p, a general RNA polymerase factor, but also by Hsf1p, a general stress response-associated transcription factor. Similar results were obtained after 48 and 72 hr of induction.

Other genes among the most strongly up-regulated (>2-fold) in the producer strain were also related to cellular responses to different stresses, including oxidative (*SUB1, GRE2, HOR2, SOD2, ATX1, HSP12, GRX5, PRX1, TSA2, GPX1, NCE103, CCP1, TRX2, AAD6*) and osmotic (*GRE2, PAI3, HOT1, HOR2, HOR7, SIP18, HSP12, RHR2*), as well as other general stress responses (*DDR2, SML1, XBP1, MSN2, HAC1, CRZ1, DAK1, YGP1, SVS1, TIR2, HSP12, ICT1, SPG4, HOR7*). Some of the genes, such as *HSP12 *(heat shock protein), appeared in multiple categories of stresses, and it was difficult to link the induction of some genes to a specific class of stress response. It needs to be further evaluated if those responses are the direct consequence of artemisinic acid and its synthesis or secondary effects. In summary, these data suggest that artemisinic acid production triggers a plethora of stress-related responses in yeast with the major classes of induced genes being the MFS and ABC-transporters.

## Discussion

The necessity for biological processes utilizing renewable resources becomes critical in part due to global warming and depletion of petroleum. Most biological engineering efforts have been focused on developing microbial systems that can be genetically altered to produce high-value chemicals and petroleum substitutes. Although our understanding of tuning microbes into efficient workhorses for chemical production is still limited, technological developments in synthetic biology promise to make biological engineering more predictable, faster, and less expensive [20]. All major classes of secondary metabolites including phenylpropanoids, terpenoids, and polyketides have been produced in microbes [7,8,13,21-25]. Yet most of the natural products are still supplied primarily through fungal fermentation and plant cultivation accompanied by subtle chemical modifications. In this respect, the yeast engineered to produce artemisinic acid is not only a step towards a potential economically viable method for producing artemisinin at a commercial scale but also a research model to study the microbial manufacture of specialty and commodity chemicals.

In this study, we demonstrated that the production titer of artemisinic acid can be increased by altering the plasmid selection marker and culture medium. Without any optimization, the artemisinic acid yields from both shake-flask and bio-reactor cultivation of the engineered yeast were 10-fold higher than those yields reported previously [7]. Further optimization of gene expression and cultivation conditions in order to take advantage of the full metabolic capacity of these microbes should improve artemisinic acid yields even more.

Although all of the amorphadiene produced was not completely oxidized to artemisinic acid, the conversion rate is estimated to be at least 32% by simply comparing the yields of amorphadiene and artemisinic acid (Figure 1B and Figure 2B). However, this value may be underestimated because the stability of *pESC-Leu2d::T *is two-fold lower than that of *pESC-Leu2d::ADS*. Considering the lower plasmid stability in artemisinic acid-producing yeast strain, the conversion rate from amorphadiene to artemisinic acid could be as high as 64%. We attempted to measure the amorphadiene that was not oxidized in the artemisinic acid-producing yeast by overlaying dodecane in the culture; however, dodecane sequesters amorphadiene efficiently and hence the total artemisinic acid production was severely impaired by dodecane (data not shown). Although almost immiscible, terpenes still have a low, but measurable water solubility. For example, a range of 5–30 mg per liter solubility in water was reported for monoterpenes such as limonene, myrcene, and pinene [26,27]. Thus, an excess of unoxidized amorphadiene is likely to remain in the medium and diffuse through the yeast membrane before being oxidized by AMO/CPR or vaporized into the atmosphere. The sequestration of amorphadiene by dodecane in cultures of artemisinic acid-producing yeast suggests that AMO/CPR inside the cell could not instantly oxidize endogenously produced amorphadiene. It is difficult to accurately measure the "in-cell conversion rate", but apparently the AMO/CPR-oxidation capability needs to be improved to fully convert the amorphadiene to artemisinic acid. Improvement of the kinetic properties of P450s is likely to be challenging, though such improvements have been demonstrated in a microbial system [28]. Nonetheless, genes/enzymes in plant secondary metabolism are evolutionarily recent, and hence there may be room for further improvement of their catalytic efficiencies by *in vitro *evolution or site-directed mutagenesis.

It was unexpected that the plasmid stability of the yeast strain expressing *ADS *in *pESC-Leu2d *reached 84% in non-selective rich medium, which was the highest of all conditions tested (Figure 1D). The plasmid stability in non-selective medium was even higher than that of yeast strain harboring the same plasmid in the selective medium. FPP and other mevalonate pathway intermediates have been reported to be cytotoxic in *E. coli *[21,29]. It is likely that yeast is also under similar stresses when FPP and other precursors accumulate inside the cells. In the yeast strain expressing *ADS *on *pESC-Leu2d *in rich medium, the expression level of *ADS *per cell could be elevated by placing it on a *Leu2d*-based high-copy plasmid and by supplying a high-level of nutrients in rich medium. Hence, potentially cytotoxic FPP could be rapidly converted to amorphadiene, which is then transported or diffuses across the plasma membrane and cell wall and evaporates from the culture medium. The reduced intracellular concentration of FPP in cells harboring a high-copy *ADS *and grown in rich medium might stabilize the plasmid harboring *ADS*. However, it should be noted that converting too much FPP to amorphadiene could also be toxic to cells as it would deplete precursors needed for ergosterol production.

The mechanism of olefin secretion from plant cells remains unknown, but hydrophobic olefins such as amorphadiene might be diffusible through the cell membrane without involving transporters. During cultivation (and production) at 30°C, amorphadiene is likely to diffuse through the membrane, and the volatile amorphadiene is readily vaporized. On the other hand, artemisinic acid produced endogenously should be ionized under neutral cytosolic conditions. Since ionized artemisinic acid requires transporters to cross the yeast plasma membrane, engineered yeast might utilize its own non-specific transporters to secrete artemisinic acid into the medium. As artemisinic acid is likely to be produced faster than it can be transported from the cell, the engineered yeast harboring the plasmid carrying *AMO *is at a selective disadvantage relative to the same yeast with no plasmid, which results in the low plasmid stabilities observed in non-selective medium (Figure 2D). This suggests that artemisinic acid might have a certain level of cytotoxicity in yeast.

The cellular stresses evoked by artemisinic acid were reflected by the massive transcriptional induction of the genes coding for ABC transporters as determined by RT-PCR and q-PCR. The unbiased microarray analysis further confirmed that the key ABC transporter genes (*SNQ2, YOR1, PDR15*, and *PDR5*) were among the genes highly up-regulated in the artemisinic acid-producing yeast. Nonetheless, yeast appears to tolerate the high-level of artemisinic acid by operating its efficient efflux pumps, such as the ABC transporters. It is difficult at this stage to identify the specific transporters or a group of transporters responsible for the secretion of artemisinic acid from yeast. In the future, it would be feasible to identify responsible transporters by deleting transporter genes in the engineered yeast or using deletion yeast strains to evaluate their sensitivity to artemisinic acid as was previously demonstrated in the artesunate (anti-malarial drug)-sensitivity test in mutant yeasts [30]. The nature and kinetic competency of yeast efflux pumps for artemisinic acid are unknown. Thus, the identification and proper expression of authentic plant artemisinic acid transporters from the plant *Artemisia annua *might enhance the efflux of artemisinic acid from engineered yeast. It would be interesting to reconstitute plant transporters in engineered yeast to see if plants have evolved an efficient transporter for a specific class of natural product such as sesquiterpenoids.

The global microarray analysis of transcriptomes of the yeast producing artemisinic acid relative to the yeast producing amorphadiene was used to identify the stress responses associated with artemisinic acid production. The microarray analysis confirmed that expression of genes encoding ABC-transporters were up-regulated in artemisinic acid-producing yeast. Additionally, microarray data indicated multiple genes involved in oxidative and osmotic stresses and other general stress-responses were significantly up-regulated. Despite the difficulties in distinguishing whether these responses were the direct effects of artemisinic acid-related toxicity or indirect effects from the initial stress responses, the T-profiler and YEASTRACT analyses revealed that at least three *cis*-elements in multiple genes were clearly responsive to artemisinic acid and/or its biosynthesis. As predicted, the Pdr3p and related transcription factors involved in multi-drug resistant signaling pathways were identified, and Msn2p/Msn4p- and Reb1p-binding motifs were also identified. The reason for the involvement of the Reb1p (RNA polymerase I enhancer binding protein) in the artemisinic acid stress is not clear, but Msn2p/Msn4p might be related to oxidative stress associated with artemisinic acid synthesis as these zinc-finger transcription factors are known to regulate superoxide dismutase and thiol peroxidase [31-33]. In the P450 catalytic cycle, reactive oxygen species (ROS) are formed when activation of molecular oxygen and substrate oxygenation are uncoupled [34]. Cytochrome P450 is a heme-thiolate enzyme in which the distal axial ligand of the heme-moiety is occupied by conserved cysteine residue [35]. The strong electron donation by anionic thiolate to the heme moiety facilitates the heterolysis of dioxygen occupying the position *trans *to the thiolate [36,37]. When cysteine was altered to glycine in the C439G AMO mutant, the heterolytic cleavage of dioxygen cannot occur, and hence ROS species are not generated.

Our microarray data are in general consistent with other yeast mutant and microarray data describing the weak acid stress-responses in yeast. The screening of gene-deletion yeasts identified *PDR5 *and *TPO1 *as major resistant determinants against the anti-malarial drug artesunate (weak acid) which is remarkably consistent with our data (*PDR5 *and *TPO1 *showed 9- and 4- fold induction at the 24 h time point; Figure 5)[30]. A comparison of yeast genes induced by treatment with 2,4-dichlorophenoxyacetic acid (2,4-D) and those by artemisinic acid produced by our engineered yeast revealed that multi-drug resistance transporters belonging to ABC and MSF transporters families (e.g. *PDR5*, *PDR15*, *YOR1 *and *TPO1*) were common genes strongly activated under both conditions [38]. Considering all data together, it is apparent that the engineered yeast is under the cellular stress exerted by a weak acid (i.e., artemisinic acid).

Finally, microarray data suggests that artemisinic acid itself may evoke osmotic stress resulting from lipophilic weak acid stress in the engineered yeast. Undissociated artemisinic acid could passively diffuse into the yeast cytosol where it dissociates into a proton and an artemisinic acid anion, which can be actively exported by the cells, resulting in an accumulation of protons in the yeast cytosol and its acidification. Alternatively, artemisinic acid could directly cause a destabilization (e.g. increased porosity) of the yeast cell wall, leading to decreased plasma membrane stability. The glycosylphosphatidylinositol (GPI)-anchored cell wall proteins, such as SPI1 and YGP1, are believed to strengthen cell walls to protect yeast against stress caused by lipophilic weak acids, such as the food preservative benzoic acid [39,40]. Induction of these genes during artemisinic acid production (approximately 1.7-fold and 4 fold, respectively) together with several other genes inducible by osmotic stress indicates that yeast tries to compensate for the membrane destabilization effect of artemisinic acid.

The fact that yeast can tolerate multiple stresses and produce one gram of artemisinic acid per liter in a laboratory scale bio-reactor suggests that yeast may be an ideal platform to produce artemisinic acid or other related lipophilic weak acids. Although the biological significance of the pleiotropic drug response and oxidative and osmotic stresses needs to be further investigated in this context and the potential resistance determinants to artemisinic acid production are still not clearly determined, this study provides a basis for endogenous artemisinic acid production-induced stresses and will be useful in selecting future potential targets for metabolic engineering.

## Conclusion

Development of microbial platforms for the production of pharmaceuticals and other industrial chemicals is an important area of research. An engineered yeast strain that synthesizes the drug precursor artemisinic acid was previously reported. We demonstrated here that the production of artemisinic acid can be significantly improved by modulating the selection marker of the expression plasmid and the composition of the culture medium. In addition, by combination of RT-PCR and microarray analysis, we showed that the cellular responses to artemisinic acid biosynthesis involve the induction of multiple pleiotropic drug resistance genes [ATP-binding cassette (ABC) and major facilitator superfamily (MSF) transporters] as well as genes known to respond to oxidative and osmotic stresses. The studies presented here provide the basis for future strain optimization to improve artemisinic acid production.

## Methods

### Plasmid construction

The Leu2d allele codes for an identical Leu2 protein sequence, but a significant portion of its promoter was deleted, leaving only a 29-bp promoter region. In order to generate pRS425-Leu2d::*ADS *plasmid, the 5'-end of Leu2-coding sequence from pRS425-Leu2::*ADS *was amplified by PCR using a pair of primers: forward primer, 5'-ACGGAGGCTTCATCGGAGATGATATCACCAAACATGTTG-3', and a reverse primer, 5'-ATGTCTGCCCCTAAGAAGATCGTCGTTTTGCCAGGTGACCACGTTGG-3'. This PCR-product was used as a template for the second PCR using a pair of primers: a forward primer, 5'-ATAACGGAGGCTTCATCGGAGATGATATCACCAAACATGTTGCTGGTGATTATAATACC-3' and a reverse primer, 5'-CGGGGCGCCTTTTTTTATATATATTTCAAGGATATACCATTGTAATGTCTGCCCCTAAGAAGATCG-3'. The deleted promoter was introduced using the reverse primer. The PCR-product was digested using *EcoRV *and *NarI *and cloned into the *EcoRV*- and *NarI*-digested pRS425-Leu2::*ADS*.

In order to generate pESC-Leu2d vector, the pESC-Leu plasmid was digested using *EcoRV *and *DraIII*, and the *EcoRV*- and *DraIII*-digested fragment from pRS425-Leu2d::*ADS *was ligated into the pESC-Leu plasmid. The *ADS *expression cassette in pRS425-Leu2d::*ADS *was amplified by using primers 5'-GTCAATCACTACGTGTAGTACGGATTAGAAGCCGCCGAGC-3' and 5'-GTCAATGCCGGCCAAATTAAAGCCTTCGAGCGTCCCA-3'. The PCR-product was digested using *DraIII *and *NaeI *and ligated into the corresponding sites in pESC-Leu2d or pESC-Leu. The coding sequence for *AMO *from pESC-URA::*AMO/CPR *was digested using *PacI *and *NotI *and ligated into the *PacI *and *NotI *of pESC-Leu2d::*ADS *and pESC-Leu::*ADS*. The coding sequence for CPR from pESC-URA::*AMO/CPR *was digested by *NotI *and *NheI *and ligated into the *NotI *and *NheI *sites of pESC-Leu2d::*ADS/AMO *and pESC-Leu::*ADS/AMO*, resulting in the plasmids, pESC-Leu2d::*ADS/AMO/CPR *and pESC-Leu::*ADS/AMO/CPR*, respectively.

### Yeast strains, cultivation and plasmid stability assay

Strains and their genotypes are given in Table 1. Strains used are derivatives of BY4742 (*MATαα his3Δ1 leu2Δ0 lys2Δ0 ura3Δ0*) [41]. The chromosomal modifications made to create strain EPY224 (BY4742 P_*GAL*1_*-tHMGR *P_*GAL*1_*-upc2-1 erg9*::*P*_*MET*3_*-ERG9 *P_*GAL*1_*-tHMGR *P_*GAL*1_*-ERG20 *plus pRS425-Leu::*ADS *and pESC-Ura::*AMO/CPR*) have been previously described [7]. Strains (EPY300) used in this study have the same genetic background as EPY224 or have repaired URA3 and LYS2 (EPY331). Repair of *ura3Δ0 *and *lys2Δ0 *in EPY300 was accomplished by transformation with a URA3 PCR amplicon (PCR primers, 5'-CTACCAGAATGATAACAAGTTTGGAC-3' and 5'-GTGGAACAGTGGTAATTCCTACGA-3'), selecting for uracil autotrophy, then transforming the resulting uracil autotroph with a LYS2 PCR amplicon (PCR primers, 5'-GGTACCTTTTTGAACTTCGTCTC-3' and 5'-CATCATGCTGCGAAGAACTAAAG-3') and selecting for lysine autotrophy. All OD_600 _(optical density at 600) measurements were taken using a Beckman DU-640 spectrophotometer. Culture tubes containing 5 mL of CSM medium with 2% glucose omitting Leu, His, and Met were inoculated with the strain of interest. These inocula were grown at 30°C to OD_600 _between 1 and 2. Unbaffled culture flasks (250 mL) containing 50 mL CSM medium omitting Leu and His or YPG medium (0.2% glucose and 1.8% galactose) supplemented with 1 mM Met were inoculated to an OD_600 _of 0.03–0.05 with the initial seed cultures for induction. For plasmid stability determination, dilutions of the cultures were plated on CSM or CSM without Leu (2% glucose) and grown for 3–4 days at 30°C before counting colonies.

### Terpenoid detection by gas chromatography-mass spectrometry

Gas chromatography (GC) and GC-mass spectrometry were used to identify and quantify amorphadiene and artemisinic acid as described previously [7].

### Semi-quantitative RT-PCR and real-time quantitative RT-PCR

Cells induced in YPG were harvested by quick centrifugation and immediately frozen in liquid nitrogen. Cells were disrupted with glass beads in a bead beater, and total RNA was extracted using the RNEasy Mini kit (QIAgen) following the manufacturer's recommendations. RNA was quantified by spectrophotometry, and its integrity verified with a 2100 Bioanalyzer (Agilent). The RNA (450 ng) was treated with the Turbo-DNA free kit (Ambion), and 350 ng of the treated RNA was reverse-transcribed with SuperScript III (Invitrogen) using an oligo(dT) primer following manufacturer's recommendations. The cDNA synthesis reactions were treated with RNAse H, diluted five fold, and 2 μl of the diluted cDNA served as template for PCR. The PCR primers used were the following: *ACT1 *(5'-GCAAACCGCTGCTCAATCTTCT-3' and 5'-CAAAGCGGTGATTTCCTTTTGC-3'); *PDR1 *(5'-ACTTACCGCAGCCCTTTGTGAG-3' and 5'-TGTCGCATGTGAAGTCCACGTA-3'); *PDR3 *(5'-TTCTCCTTGCCCCATCAAGAAA-3' and 5'-CTCCCAACGAGACAATCCCATC-3'); *PDR5 *(5'-TTTTCTAGTGCCGCCTGGGTTA-3' and 5'-CACCTGGGTTTAGGCAACCATC-3'); *PDR12 *(5'-TGTCCCGAGCTTGATTTCCATT-3' and 5'-CCTCTTGTGGCGTTATCCCAAG-3'); *PDR15 *(5'-TGTTGGTGTTGCAGGTGAAGGT-3' and 5'-AGGGTTTGCATCAGGTGGACAT-3'); *SNQ2 *(5'-AACCGCGTTGACTGATCCAAAT-3' and 5'-AAAAGCACCGGAAGTGGATGAA-3'); and *YOR1 *(5'-GGAGGCTCCAGAAGATGATCCA-3' and 5'-CTAAATCTTCCGGGCCGTCTCT-3'). For semi-quantitative PCR, reactions were conducted for the indicated number of cycles, and products were visualized on agarose gels using standard techniques. For quantitative PCR, 2X master mixes (DyNAmo HS SYBR Green qPCR kit, Finnzymes or iQ SYBR Green Supermix, Bio-Rad) were used according to manufacturer's recommendations on a Bio-Rad iCycler instrument. Three independent transformants of each strain were assayed in triplicate (n = 9), and the coefficient of variation of C_T _for all replicate measurements was < 3%. Relative gene expression was calculated using the Pfaffl method [42] with *ACT1 *as the reference transcript.

### Microarray analysis

Five independent transformants of each strain were induced in YPG and harvested by centrifugation after 24, 48 and 72 h of induction and cell pellets were immediately frozen in liquid nitrogen. Total RNA was prepared by hot acid phenol extraction [43] and further purified on an RNEasy mini column (QIAgen). The RNA integrity was verified using a 2100 Bioanalyzer (Agilent). Equal amounts of total RNA from five clones of each strain were pooled. Twenty (20) μg of each sample was used for reverse transcription and fluorescent labeling with SuperScript Plus Indirect cDNA Labeling System (Invitrogen) following the manufacturer's recommendations, except for the chemical hydrolysis of RNA and cDNA purifications, which were carried according to standard protocols . Labeled cDNA targets were hybridized to cDNA microarray slides double-spotted with the 6,240 predicted ORFs of *S. cerevisiae *(University Health Network Microarray Facility, Toronto, Canada) and washed using standard methods on a hybridization station (TECAN). Images were acquired with a GenePix Professional 4200A scanner (Axon Instruments), and feature fluorescence intensities were extracted with the GenePix Pro 6.1 software. Four or five replicates slides for each time point were hybridized. Artifactual or aberrant spots were excluded, as well as spots with low signal in both channels and spots for which more than 50% of the values were missing. Lowess normalization was performed using BRB-ArrayTools version 3.5.0 developed by Dr. Richard Simon and Amy Peng Lam . Significant differentially expressed genes were extracted using the SAM software [44], with false discovery rate set at ≤ 0.2%. Clustering and gene ontology analyses were performed on the significantly differentially expressed genes using Genesis 1.7.1 [45].

### Bioreactor

The fermentation was carried using a Sartorius Biostat B+ bioreactor. The temperature was kept at 30°C and pH was kept at 5.5 with addition of 4 M NaOH. The OD was monitored using an Optek ASD19-N single channel NIR absorption probe and the off-gas analysis was monitored by a Thermo Onix VG Prima δB mass-spectrometer. The yeast was firstly grown in batch fermentation with 27 g L^-1 ^initial glucose concentration, vitamins and trace metals solution in an initial volume of 720 ml. When glucose was depleted after approximately 21 h, a solution of 186 g L^-1 ^of glucose, vitamins and minerals started to be added in the bioreactor with feed-rate addition controlled by a D.O stat algorithm [46]. After 40 h, the OD reached approximately 60 and the broth volume in the bioreactor was approximately 920 ml. At this point, production was induced by another feed solution containing 377 g L^-1 ^of galactose, vitamins and trace metals which was added into the bioreactor for 72 h at a feed rate also controlled the D.O. stat.

## Authors' contributions

DKR, MO, EMP, CP, JDN, and JDK designed experiments. DKR, MO, and EMP conducted plasmid construction, analytical chemistry, and molecular biology. DE and CP conducted yeast genetics. HB conducted the fermentation experiment. All authors contributed to the manuscript writing.

## Supplementary Material

Additional file 1 **A list of genes significantly differentially expressed in EPY330 or EPY338**. The additional data file includes a processed data set for differentially expressed genes identified by microarray analysis. The data set has 2,156 genes that were significantly differentially expressed in at least one experimental time points and has 488 and 256 genes that were up-regulated and down-regulated, respectively, in the producer strain by more than 2-fold at least one time points. Click here for file
